# Transcription profiles of chicken liver and spleen in response to infection with avian pathogenic *Escherichia coli* at different stages

**DOI:** 10.1016/j.psj.2026.106579

**Published:** 2026-02-02

**Authors:** Tailong Wang, Yifei He, Tingyu Zhang, Yan Luo, Mengru Chen, Chu Meng, Haitao Zhang, Ruqian Zhao, Yimin Jia

**Affiliations:** a Key Laboratory of Animal Physiology & Biochemistry, College of Veterinary Medicine, Nanjing Agricultural University, Nanjing, Jiangsu 210095, PR China; b MOE Joint International Research Laboratory of Animal Health & Food Safety, Nanjing Agricultural University, Nanjing, Jiangsu 210095, PR China; c Jiangsu Lihua Foods Group Co., Ltd., PR China

**Keywords:** Avian pathogenic *Escherichia coli*, Broiler, Liver, Spleen, Transcription

## Abstract

Avian pathogenic *Escherichia coli* (**APEC**) is an extraintestinal pathogen that causes diverse local and systemic infections in poultry and poses a potential zoonotic threat. Although extensive research has addressed its antimicrobial resistance, virulence factors, and host immune responses, the temporal transition from early infection to recovery remains poorly characterized. Here, we established a systemic infection model in broilers by inoculating the pectoral muscle with *E. coli* O18:K1 and monitored sequential changes in organ weights and indices, serum biochemistry, and histopathology. Parallel RNA-sequencing of liver and spleen tissues delineated stage-specific transcriptional reprogramming. Peak mortality occurred 2 days post-infection (**dpi**) and ceased at 5 dpi. The infected and uninfected control groups differed significantly in organ weights and indices and blood biochemical indicators at 2 dpi; however, many indicators recovered by 5 dpi in the infected group. Liver transcriptomics during acute infection revealed concerted downregulation of minichromosome maintenance (**MCM**) family genes and concurrent suppression of the cell cycle and DNA repair pathways. By 5 dpi, these genes and pathways were reactivated, mirroring the transition from pathology to recovery. Key immune mediators in the spleen (*IL1B, IL6, FOS, PTGS2, JUN*, and *NFKBIA*) were enriched in immune response pathways and displayed a temporal switch from activation to repression, underscoring the dynamic regulation imposed by this immune organ. Collectively, our data provide a comprehensive spatiotemporal map of the molecular and immune landscape that demarcates the infection and recovery phases of APEC in broilers.

## Introduction

Avian pathogenic *Escherichia coli* (**APEC**) is an extraintestinal pathogen that causes diverse local and systemic infections in birds and humans, resulting in extensive economic losses to the poultry industry worldwide (Mehat et al., 2021; Monson and Lamont, 2021). Considering the heterogeneity in virulence and serotypes, and the widespread multidrug resistance among pathogenic *E. coli* variants (Nawaz et al., 2024), traditional prophylactic and therapeutic measures face critical limitations, making improvements in genetic resistance to disease and immunocompetence in poultry an effective alternative (Alber et al., 2021). However, the systemic defense responses of broilers to APEC infection remain poorly understood.

APEC colonizing the respiratory tract disseminates through the bloodstream to vital organs (e.g., spleen and liver), causing severe tissue damage and dysregulated inflammation that might progress to sepsis, multiple-organ failure, and mortality (Hu et al., 2022). The spleen is a pivotal immune organ that filters blood-borne pathogens and facilitates antigen presentation, thereby orchestrating rapid, lymphocyte-driven responses (Lewis et al., 2019). Previous studies comparing spleen transcriptomics between mild and severe pathological groups following APEC infection revealed *CD148, CD45*, and lymphocyte cell-specific protein-tyrosine kinase (***LCK***) as key candidate genes in T cell receptor signaling (Nie et al., 2012), while implicating Toll-like receptor, Jak-STAT, and cytokine signaling as critical signaling pathways (Sandford et al., 2011). Shi et al. (2023) analyzed proteomic and transcriptomic data from spleens post-APEC infection, revealing that differentially expressed proteins were significantly enriched in the phagosome and lysosome pathways.

The liver functions as a pivotal immunological organ in which myeloid cells, including Kupffer cells and neutrophils, orchestrate systemic immune defense by clearing bacteria, secreting acute-phase proteins and cytokines, and modulating metabolic processes in response to inflammation (Strnad et al., 2017). Notably, APEC infection can induce hepatocellular degeneration (Sadeyen et al., 2014), inflammatory cell infiltration, and elevate liver damage indicators in the blood (Kumari et al., 2020). However, these studies predominantly focused on organ-confined pathogenic mechanisms during the early phase of infection, leaving the inter-organ immune crosstalk that orchestrates systemic APEC pathogenesis largely unexplored.

To address this knowledge gap, we systematically characterized the spatiotemporal transcriptional landscape of liver and spleen tissues during APEC infection, aiming to identify resistance-associated pathways and candidate genes in broilers. We hypothesized that the host response is governed by temporally dynamic and organ-specific immune programs. This investigation could provide mechanistic insights into the intricate regulatory networks orchestrating avian innate immune responses during systemic bacterial challenge.

## Materials and methods

### Ethical statement

All procedures used in this study were approved by the Institutional Animal Care and Use Committee of Nanjing Agricultural University (approval number NJAU.No20240517094) and followed the Guidelines on Ethical Treatment of Experimental Animals (2006) No. 398 set by the Ministry of Science and Technology (2006, Beijing, China).

### Bacteria

The *E. coli* O18:K1 strain was isolated from birds clinically diagnosed with pericarditis and perihepatitis and preserved at Jiangsu Lihua Foods Group Co., Ltd. The strain was grown in Luria–Bertani (**LB**) broth at 37°C in a thermostatic incubator until reaching an optical density of approximately 0.6 at 600 nm. *E. coli* O18:K1 bacteria were collected by centrifugation and resuspension in sterile saline using a serial dilution protocol to estimate viable counts. The bacteria were preserved in glycerol stock at –80°C.

### Experimental design

A total of 300 healthy 1-day-old male broilers were purchased from a commercial hatchery (Changzhou Siji Poultry Industry Co., Ltd., China). For survival curve analysis, 100 broilers were randomly assigned to uninfected control or challenged groups (n = 50 per group), with cumulative mortality recorded throughout the 14-day observation period. The remaining 200 broilers were designated as uninfected control (*n* = 50) and infected (*n* = 150) groups for subsequent analyses. Broilers in the infected group received a 0.2 mL intramuscular injection of *E. coli* O18:K1 suspension (5 × 10^6^ CFU/mL in sterile saline), whereas those in the uninfected control group were administered an equivalent volume of sterile saline via the same route. Broilers were housed in isolation units under controlled ventilation and temperature, with free access to water and feed. At 2 and 5 dpi, 12 broilers per group were randomly selected and euthanized. Subsequently, several organs (heart, liver, spleen, and bursa of Fabricius) were dissected and weighed, and the organ index for each organ was calculated as the ratio of organ weight to body weight. Histopathological analysis was performed on tissues from the left hepatic lobe and central spleen (*n* = 4 per group); corresponding samples from additional broilers were snap-frozen in liquid nitrogen for transcriptomic analysis (*n* = 5) . The blood samples were centrifuged at 4°C and 3,000 × *g* for 10 minutes. Both tissues and plasma were stored at –80°C pending analysis.

### Histopathological analysis

Paraformaldehyde-fixed liver and spleen samples were dehydrated, embedded in paraffin, and manually sectioned with a microtome to obtain 4–5 μm-thick paraffin sections. The dewaxed sections were stained with hematoxylin and eosin, and images were acquired using a light microscope (Olympus BX53, Japan).

### Blood biochemistry

Plasma biochemical parameters were measured to evaluate liver injury [alanine aminotransferase (**ALT**), aspartate aminotransferase (**AST**), alkaline phosphatase (**ALP**), and lactate dehydrogenase (**LDH**)], systemic energy metabolism [glucose (**Glu**), cholesterol (**CHOL**), and triglycerides (**TG**)], oxidative stress [superoxide dismutase (**SOD**)], immune-related protein metabolism [total protein (**TP**), albumin (**ALB**), and globulin (**GLOB**)], and serum iron status during APEC infection. All assays were performed on an automatic biochemistry analyzer (Hitachi 7020, Japan) using commercial kits (Medicalsystem Biotechnology Co., Ltd., Ningbo, China) according to the manufacturer’s instructions. GLOB concentration was calculated as TP minus ALB.

### RNA isolation, library construction, and sequencing

Total RNA was extracted using TRIzol reagent and assessed for quality and quantity using the RNA Nano 6000 assay kit and an Agilent Bioanalyzer 2100. Five biological replicates per group were processed for RNA-seq analysis to ensure paired liver and spleen samples from each chicken. Three spleen samples from the uninfected control group were excluded as they failed the RNA integrity criteria during quality control. Qualified RNA samples were used to construct libraries with a MGIEasy RNA library preparation kit. The mRNA was enriched using oligo(dT)-coated magnetic beads, fragmented, and reverse-transcribed into double-stranded cDNA. The cDNA underwent end repair, single-dA extension, adapter ligation, library amplification, and purification. Sequencing was performed on the DNBSEQ-T7 platform, generating 150 bp paired-end reads. The RNA-seq data generated in this study have been deposited in the NCBI Gene Expression Omnibus (**GEO**) database under accession number GSE282645.

### Differential expression and functional enrichment analyses

The raw data in FASTQ format were filtered with fastp (v0.20.1). The clean reads of each sample were aligned to the reference genome GRCg7b using HISAT2 (v2.2.1) software to obtain raw counts of reads aligned to each gene. The original counts were converted to transcripts per million (**TPM**) values using a Python script. The top 5% of differentially expressed genes (**DEGs**) were selected for z-score normalization, helping visualize the core transcriptional programs during APEC infection (Li et al., 2022). Subsequently, hierarchical clustering was performed using the scikit-learn (v1.5.1) library, followed by Gene Ontology (**GO**) enrichment analysis of each cluster on the DAVID platform. Spearman correlations were calculated among the various liver and spleen groups. Transcripts with a threshold of |log2 fold change| > 1 and *p* < 0.05 were identified as differentially expressed using DESeq2. Enrichment analysis of upregulated and downregulated genes was performed using the Kyoto Encyclopedia of Genes and Genomes (**KEGG**) on the DAVID platform and visualized in R.

### Construction of candidate gene network and function analysis

Genes identified by the KEGG pathway analysis as specifically enriched at either the acute (2 dpi) or recovery (5 dpi) stage—encompassing 27 pathways in the liver and 21 in the spleen—were submitted for targeted analysis. Proteins predicted via the STRING database (https://string-db.org/, accessed 19 October 2024) for stage-specific genes were used to construct protein–protein interaction (**PPI**) networks. The top 10 candidate genes within these networks were identified using the cytoHubba plug-in in Cytoscape, based on the degree algorithm, to pinpoint central drivers of the host response to APEC infection (Chin et al., 2014; Shannon et al., 2003).

### Statistical analysis

The data were analyzed using IBM SPSS Statistics for Windows, version 27.0 (IBM Corp., Armonk, NY, USA). Continuous variables are presented as mean ± SEM and were compared using the Mann–Whitney *U* test. Statistical significance was set at *P < 0.05 and **P < 0.01. Kaplan–Meier survival curves were generated using GraphPad Prism 10, and R packages were used for DEG analysis.

## Results

### Mortality rate and organ weights

No deaths were recorded in the uninfected control group throughout the trial period. In contrast, APEC infection led to an overall mortality rate of 76% (38/50 broilers). The highest mortality occurred on 2 dpi (24%, 12/50), followed by continued deaths from 3 to 5 dpi (52%, 26/50). Notably, no further deaths were observed between 5 and 14 dpi, indicating that the infection entered a recovery phase and the condition of surviving broilers stabilized (Fig. S1).

**Fig. 1:**
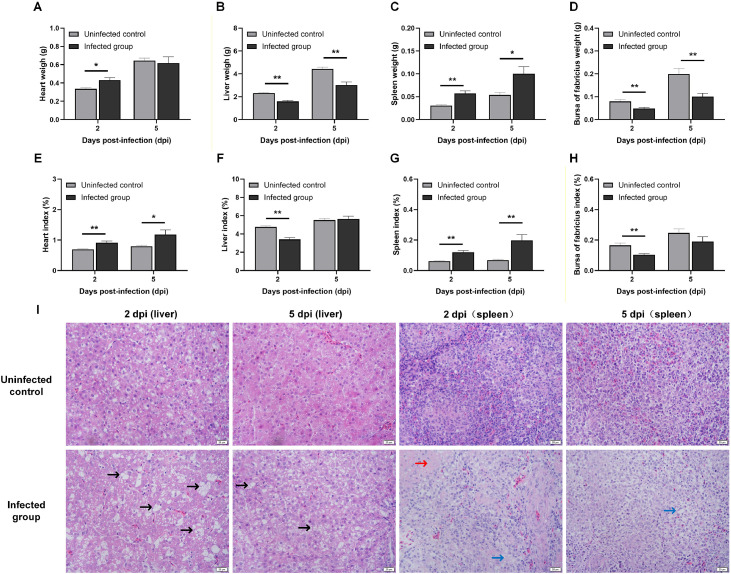
Pathological alterations of broilers challenged with APEC.

At 2 dpi (the acute phase), broilers with APEC infection had significantly higher heart (Fig. 1A, *P* < 0.05; Fig. 1E, *P* < 0.01) and spleen (Fig. 1C, *P* < 0.01; Fig. 1G, *P* < 0.01) weights and indices and significantly lower liver (Fig. 1B, *P* < 0.01; Fig. 1F, *P* < 0.01) and bursa of Fabricius (Fig. 1D, *P* < 0.01; Fig. 1H, *P* < 0.01) weights and indices, respectively, than broilers in the uninfected control group. The pattern changed markedly by 5 dpi (recovery phase), with only spleen weight (Fig. 1C, *P* < 0.01) and heart (Fig. 1E, *P* < 0.01) and spleen (Fig. 1G, *P* < 0.01) indices remaining significantly elevated, and liver (Fig. 1B, *P* < 0.01) and bursa of Fabricius (Fig. 1D, *P* < 0.01) weights remaining significantly decreased in the infected group. Notably, the significant reductions in liver and bursa of Fabricius indices observed at 2 dpi, were no longer present at 5 dpi.

(A) Heart weight; (B) Liver weight; (C) Spleen weight; (D) Bursa of Fabricius weight; (E) Heart index; (F) Liver index; (G) Spleen index; (H) Bursa of Fabricius index. (I) Histopathology of liver and spleen structures in broilers challenged at 2 dpi and 5 dpi. The results are presented as the mean ± SEM, n = 4; * P < 0.05, ** P < 0.01. Vacuolar degeneration of liver cells can be seen in the liver (black arrow). Lymphocyte reduction (blue arrow) and sero-fibrinous exudation (red arrow) can be observed in the spleen. Scale bar = 20 µm, n = 3.

### Histopathological lesions in the liver and spleen

**Fig. 2:**
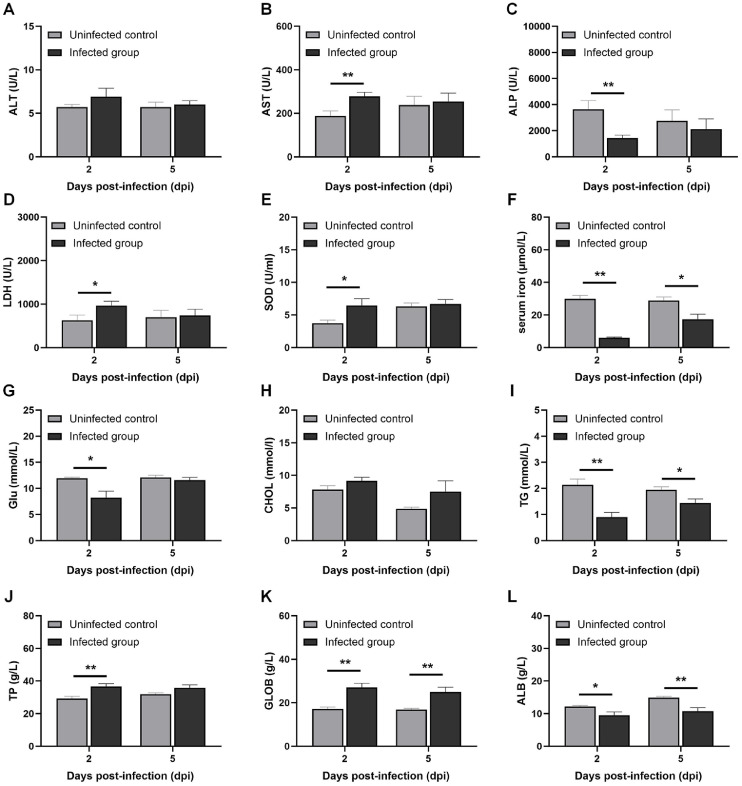
Blood biochemical indicators of broilers challenged with APEC.

Histopathological analysis revealed distinct tissue-specific injury and repair processes (Fig. 1I). In the liver, APEC infection led to evident hepatocellular apoptosis and necrosis at 2 dpi, along with disordered arrangement of liver cords, enlargement of liver sinusoidal spaces, hepatocyte rupture, and nuclear dissolution. The hepatic chord structure was markedly clearer at 5 dpi, with only a small amount of vacuolar degeneration observed. Furthermore, enlarged interstitial spaces, serofibrinous exudation, leukocytic infiltration of the capsule, and lymphocyte depletion were observed at 2 dpi in spleens from the infected group, with apparent lymphocyte necrosis at 5 dpi (Fig. 2).

(A) Plasma ALT activity; (B) Plasma AST activity; (C) Plasma ALP activity; (D) Plasma LDH activity; (E) Plasma SOD activity; (F) Plasma serum iron concentrations; (G) Plasma Glu concentrations; (H) Plasma CHOL concentrations; (I) Plasma TG concentrations; (J) Plasma TP concentrations; (K) Plasma GLOB concentrations; (L) Plasma ALB concentrations. The results are presented as the mean ± SEM, n = 8; * P < 0.05, ** P < 0.01.

### Blood biochemical indicators

**Fig. 3:**
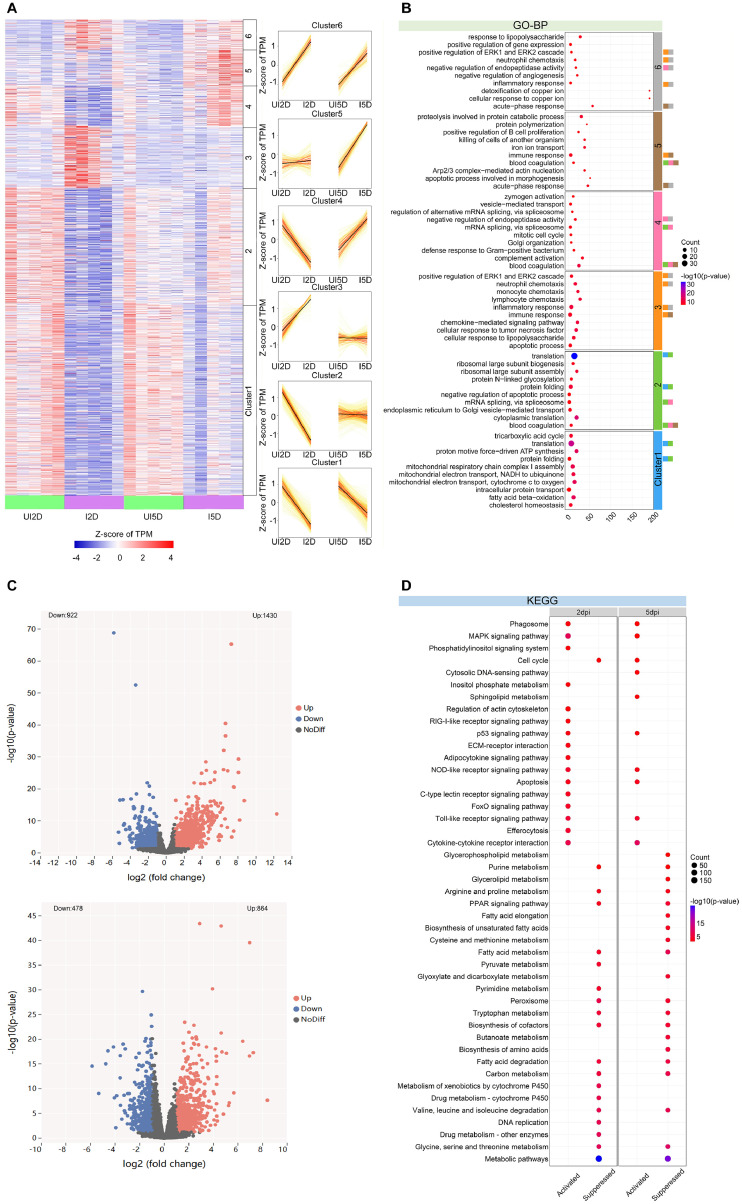
The transcriptomic profiles of liver in broilers challenged with APEC*.*

At 2 dpi, APEC infection significantly elevated plasma enzyme activities of AST (Fig. 3B, *P* < 0.01), LDH (Fig. 3D, *P* < 0.05), and SOD (Fig. 3E, *P* < 0.05), as well as plasma concentrations of TP (Fig. 3J, *P* < 0.01) and GLOB (Fig. 3K, *P* < 0.01). Conversely, it significantly decreased ALP activity (Fig. 3C, *P* < 0.01) and plasma levels of iron (Fig. 3F, *P* < 0.01), Glu (Fig. 3G, *P* < 0.05), TG (Fig. 3I, *P* < 0.01), and ALB (Fig. 3L, *P* < 0.05). By 5 dpi, most altered parameters had returned to levels comparable with those in the uninfected control group. However, plasma iron (Fig. 3F, *P* < 0.05), TG (Fig. 3I, *P* < 0.05), and ALB (Fig. 3L, *P* < 0.01) remained significantly decreased, while GLOB concentration (Fig. 3K, *P* < 0.05) remained significantly increased in the infected group.

(A) Differential gene expression of six clusters revealed by *k*-means clustering. Left: expression heatmap drawn using Z-score of TPM values for each gene in the six clusters. Right: expression profiles of the six clusters. The black lines represent mean gene expression levels, and the orange lines represent gene expression levels for each gene in the relative cluster during infection; (B) The top ten significantly enriched gene ontology biological process (GO-BP) terms for genes in each cluster. Distinct colors denote different functional clusters, with shared GO terms indicated by multiple-colored boxes; (C) Volcano plots of DEGs in the liver at 2 dpi (top) and 5 dpi (bottom) in the infected group compared with the uninfected control; (D) The activation and suppression of KEGG pathways in the liver at 2 dpi and 5 dpi. UI2D, uninfected group at 2 dpi; I2D, infected group at 2 dpi; UI5D, uninfected group at 5 dpi; I5D infected group at 5 dpi.

### Liver transcriptomic profiles in broilers

Sequencing generated 100.4 Gb of high-quality bulk RNA-seq data from five biological liver replicates in each group. There were 11,443 evidently expressed genes [transcripts per million (TPM) > 0.5 in at least three replicates for a given group] in the liver during APEC infection.

To evaluate transcriptional homogeneity within experimental groups and identify core transcriptional programs, we analyzed sample correlations and identified the top 5% most highly expressed genes in the liver. Spearman correlation analysis among all 20 samples revealed strong intra-group consistency (r = 0.821–0.991; Fig. S2A). Correlations between the infected and uninfected groups were lower at 2 dpi (r = 0.605–0.905; Fig. S2A) but increased at 5 dpi (r = 0.843–0.923; Fig. S2A), indicating a transition from the acute to the recovery phase. Among the highly expressed genes (top 5%), three-quarters (424 genes) were consistently shared across all four groups (Fig. S2B). These genes were primarily enriched in basal biological processes such as translation, cytoplasmic translation, and proton-motive force-driven ATP synthesis. Stage-specific functional shifts were also observed: the infected group at 2 dpi (I2D) group was enriched for terms related to the antimicrobial humoral immune response mediated by antimicrobial peptides, whereas the I5D group exhibited unique enrichment in intracellular iron ion homeostasis (Fig. S2C). This functional transition reflects a coordinated shift in the liver from an active inflammatory response at 2 dpi to a state of metabolic and ion homeostasis at 5 dpi.

Investigation of gene expression during infection identified six major patterns (Fig. 3A). Compared with the uninfected control group, Clusters 1, 2, and 4 were downregulated at 2 dpi. Whereas Cluster 2 recovered at 5 dpi, Cluster 1 remained downregulated, and Cluster 4 became upregulated. Functional enrichment analysis using GO-BP showed that Clusters 1 and 2 were mainly involved in protein synthesis and energy metabolism, including translation, protein folding, and the tricarboxylic acid cycle (Fig. 3B). Cluster 4 was primarily associated with complement activation and defense responses to Gram-positive bacteria. In contrast, Clusters 3 and 6 exhibited upregulation at 2 dpi. Whereas Cluster 3 reverted to uninfected levels at 5 dpi, Cluster 6 remained upregulated. Genes in these clusters were predominantly involved in immune-related processes (e.g., inflammatory cell chemotaxis and apoptosis process), indicating that the infected group mounted a rapid response to pathogen invasion.

Compared with the UI2D group, 1,430 genes were significantly upregulated, and 922 were downregulated in the I2D group (Fig. 3C). Compared with the UI5D group, 864 genes were significantly upregulated and 478 were downregulated in the I5D group (Fig. 3C).

KEGG pathway analysis revealed that APEC infection significantly upregulated hepatic immune signaling pathways at 2 dpi, notably the C-type lectin receptor signaling pathway and the MAPK signaling pathway, while concomitantly downregulated key cellular metabolism pathways, including those related to cell cycle progression, DNA replication, and fatty acid metabolism (Fig. 3D). Conversely, immune pathway activity was attenuated, and cell cycle progression exhibited significant reactivation at 5 dpi.

### Spleen transcriptomic profiles in broilers

Sequencing generated 89.2 Gb of high-quality bulk RNA-seq data from five biological spleen replicates at each stage. There were 13,089 evidently expressed genes (TPM > 0.5 in at least three replicates for a given group) in the spleen during APEC infection.

Spearman correlation analysis of all 17 samples revealed strong intra-group consistency (r = 0.821–0.994; Fig. S2D). Correlations between the infected and uninfected groups were lower at 2 dpi (r = 0.670–0.844; Fig. S2D) but increased at 5 dpi (r = 0.718–0.926; Fig. S2D), indicating a transition from the acute to the recovery phase. Among the most highly expressed genes (top 5%), three-quarters (491 genes) were consistently shared across all four groups (Fig. S2E). These genes were primarily enriched in basal biological processes essential for cellular maintenance, including translation, cytoplasmic translation, and proton-motive force-driven ATP synthesis. Beyond these shared functions, stage-specific signatures emerged: the I2D group was uniquely characterized by cellular responses to lipopolysaccharide and immune response, whereas the I5D group exhibited a specific focus on mitochondrial electron transport (Fig. S2F).

**Fig. 4:**
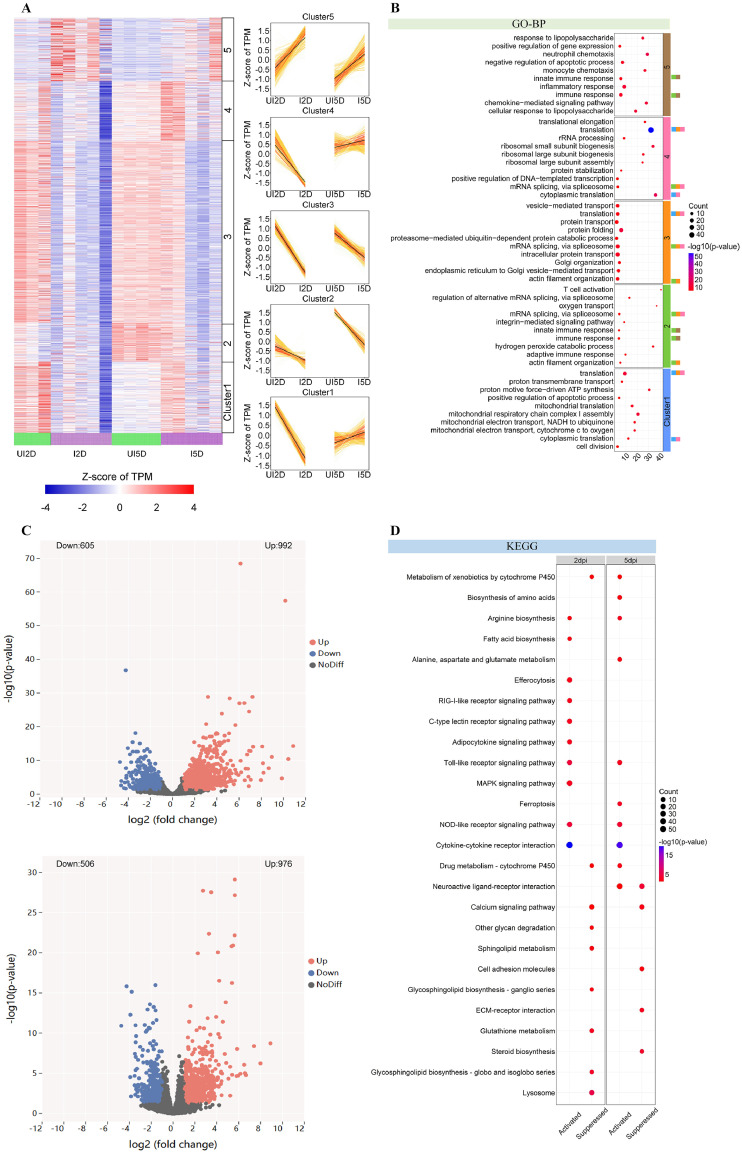
The transcriptomic profiles of spleen in broilers challenged with APEC.

Investigation of gene expression during infection identified five major patterns (Fig. 4A). Compared with the uninfected control group, Clusters 1, 2, 3, and 4 were downregulated at 2 dpi in the infected group. Whereas Clusters 2 and 3 remained downregulated at 5 dpi, Clusters 1 and 4 became upregulated. Functional enrichment analysis using GO-BP showed that Clusters 2 and 3 were mainly involved in protein synthesis and mRNA splicing processes, including translation and mRNA splicing via the spliceosome (Fig. 4B). Clusters 1 and 4 were primarily associated with protein synthesis and ribosome assembly. Conversely, Cluster 5 was upregulated at both 2 and 5 dpi. Genes in Cluster 5 were predominantly associated with immune-related processes (e.g., inflammation and immune responses), indicating that the infected group mounted a sustained defense against pathogen invasion.

(A) Differential gene expression of six clusters revealed by *k*-means clustering. Left: expression heatmap drawn using Z-score of TPM values for each gene in the five clusters. Right: expression profiles of the five clusters. The black lines represent mean gene expression levels, and the orange lines represent gene expression levels for each gene in the relative cluster during infection; (B) The top ten significantly enriched gene ontology biological process (GO-BP) terms for genes in each cluster; (C) Volcano plots of DEGs in the spleen at 2 dpi (top) and 5 dpi (bottom) in the infected group compared with the uninfected control; (D) The activation and suppression of KEGG pathways in the spleen at 2 dpi and 5 dpi.

Compared with the UI2D group, 992 genes were significantly upregulated and 605 were downregulated in the I2D group (Fig. 4C). Compared with the UI5D group, 976 genes were significantly upregulated and 506 were downregulated in the I5D group (Fig. 4C).

KEGG pathway analysis revealed that APEC infection significantly activated immune signaling pathways in the spleen at 2 dpi, including the C-type lectin receptor signaling pathway and the MAPK signaling pathway. Concurrently, metabolism- and signaling-related pathways, such as those related to glutathione metabolism, lysosome, and neuroactive ligand–receptor interaction, were notably inhibited (Fig. 4D). The numbers of activated immune signaling pathways and inhibited metabolic pathways decreased at 5 dpi.

### Construction of PPI networks and identification of candidate genes

To delineate phase-specific molecular determinants, we extracted genes from KEGG pathways uniquely enriched at the acute (2 dpi) *versus* the recovery (5 dpi) stage (27 liver and 21 spleen pathways) and constructed corresponding PPI networks using the STRING database. The top 10 hub genes were identified using the cytoHubba plug-in in Cytoscape, based on the degree algorithm, as central regulatory nodes of the host response to APEC infection.

**Fig. 5:**
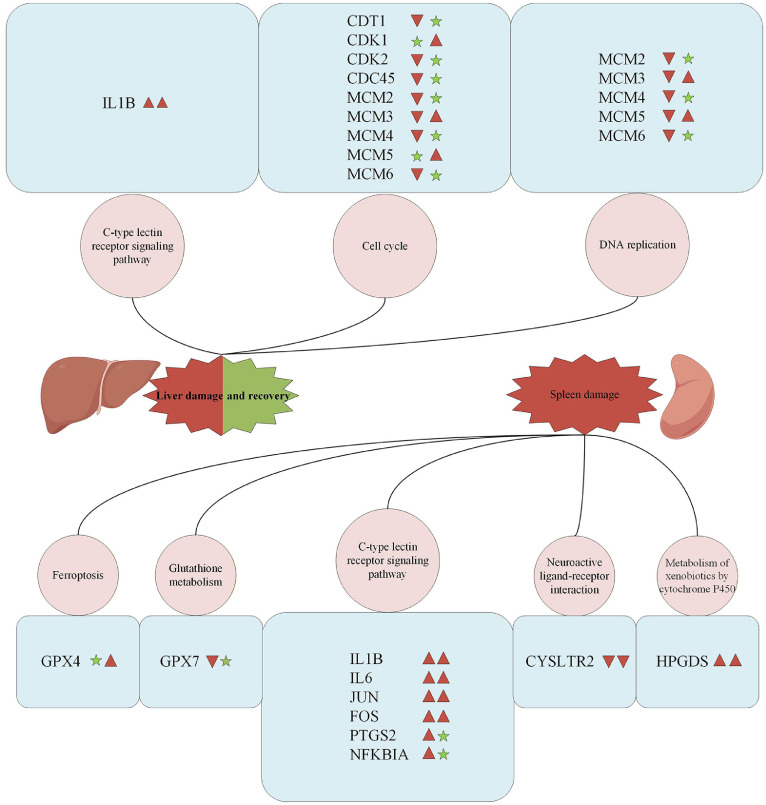
The functional network diagrams of candidate genes for the liver and spleen.

In the liver, this analysis highlighted a robust cluster of genes including *MCM2/MCM3/MCM4/MCM5/MCM6,* chromatin licensing and DNA replication factor 1 (***CDT1)***, cyclin dependent kinase 1 (***CDK1***), *CDK2*, and cell division cycle 45 (***CDC45***), all primarily involved in cell cycle progression, as well as the key inflammatory mediator *IL1B* (Fig. 5). Notably, these candidate genes exhibited distinct stage-specific expression patterns, suggesting a coordinated shift in which the host balances intensive cellular proliferation with modulation of inflammatory signals during the transition from acute infection to recovery.

KEGG signaling pathway maps are enclosed by the pink circle. Candidate genes are highlighted within the blue box. Among these, red upward triangles indicate upregulation, red downward triangles indicate downregulation, and green five-pointed stars indicate no significant difference.

In the spleen, hub gene analysis identified a distinct set of regulatory nodes that differed from those in the liver. Compared with the uninfected control group, the infected group displayed stage-specific shifts in a cluster of core inflammatory mediators, including *IL1B, IL6*, Fos proto-oncogene AP-1 transcription factor subunit (***FOS***), jun proto-oncogene (***JUN***), NF-kappa-B inhibitor alpha (***NFKBIA***), and prostaglandin-endoperoxide synthase 2 (***PTGS2***) and hematopoietic prostaglandin D synthase (***HPGDS***; Fig. 5). Additionally, the identification of glutathione peroxidase 4 (***GPX4***), *GPX7*, and cysteinyl leukotriene receptor 2 (***CYSLTR2***) as hub genes suggests that the splenic response is highly focused on modulating oxidative stress, ferroptosis and lipid-derived inflammatory signals.

## Discussion

*Escherichia coli* infection during the first post-hatch week critically impairs broiler performance by reducing weight gain, impairing immune organs, and decreasing the birds’ weight homogeneity, thereby threatening profitability (Swelum et al., 2021). Many studies have employed intratracheal inoculation to recapitulate the clinicopathological manifestations of natural APEC infection, including pericarditis, perirenal inflammation, and airsacculitis (Li et al., 2025b; Lun et al., 2025). In this study, intramuscular inoculation recapitulated the clinicopathological manifestations of APEC infection while inducing an earlier mortality peak and a shorter disease course relative to respiratory challenge (Ye et al., 2025), suggesting that intramuscular injection could be a robust model for investigating APEC-triggered inflammatory and immune responses.

Organ weights and indices are critical indicators of avian immunocompetence (Jie et al., 2025). Our study revealed that both spleen weight and index were significantly higher in the infected group at 2 dpi, consistent with previous reports (Li et al., 2025a). These alterations conformed with histopathological evidence of splenic leukocyte infiltration at 2 dpi and subsequent attenuation at 5 dpi, suggesting that the spleen orchestrates antimicrobial defense via leukocyte recruitment during acute infection and restores homeostasis during lesion resolution in the recovery phase. However, whereas our liver weight and index findings diverged from those reported by Song et al. (2022), hepatocellular necrosis on histopathology were consistent with prior reports (He et al., 2019; Wang, 2014). We speculated that different modes of infection may be related to the varying degrees of inflammatory damage to the liver.

Blood biomarkers are objectively measured parameters used to assess systemic pathophysiological changes during infection, providing direct insights into organ damage and metabolic homeostasis and predicting disease prognosis (Kaushik et al., 2025; Xu et al., 2025). In this study, APEC infection decreased plasma Glu and TG levels at 2 dpi, with only Glu returning to normal by 5 dpi. Our observations were consistent with those reported by Zha et al. (2025). This phenomenon likely arises from nutritional deficits induced by infection-associated anorexia, coupled with immune-mediated nutrient repartitioning (Kedia-Mehta and Finlay, 2019). Moreover, we evaluated physiological blood parameters, including liver injury markers and acute-phase immune responses, during APEC infection. Prior studies have documented marked increases in plasma AST, LDH, and SOD levels following APEC infection, indicating acute liver injury (Chen et al., 2025a; Hashem et al., 2022). Our findings corroborated these observations. Additionally, hypoalbuminemia and decreased plasma ALP activity were associated with poor prognosis in patients with bacterial and fungal infections (Kanai et al., 2008; Pikoulas et al., 2026). This was corroborated by survival curve analysis, which demonstrated progressive mortality in approximately 50% of survivors beyond 2 dpi.

Iron is crucial for the growth, metabolism, and reproduction of most bacteria, including APEC; however, it is often limited in the host environment, leading to competition for iron between APEC and broilers during infection (Nawaz et al., 2024). Tufft and Nockels (1991) reported that *E. coli* infection significantly depleted host serum iron levels, consistent with our results. The liver and spleen, as the largest iron reserves, play an important role in iron competition during host defense against pathogens (Mu et al., 2021). In this study, we found that the liver stimulated iron ion transport and detoxified copper–iron to counter bacterial invasion, while the spleen uniquely activated ferroptosis to deplete lymphocytes during immune responses, in line with our histological observations. These differential responses likely reflect the distinct cellular composition and functional organization of hepatic and splenic tissues (Floreste et al., 2023).

The spleen is the primary site for specific immune responses and adaptive immunity (Alexandre and Mueller, 2023), whereas the liver functions as a metabolic hub that harbors the body’s largest macrophage reservoir in the form of Kupffer cells (Strnad et al., 2017). Analysis of highly expressed gene clusters revealed that APEC infection elicited an acute-phase inflammatory response in the liver while suppressing mitochondrial fatty acid oxidation and cholesterol metabolism. Temporal profiling demonstrated attenuated inflammatory cell chemotaxis and diminished apoptosis in the liver, concomitant with progressive restoration of protein translation, complement activation and initiation of mitosis. Conversely, the spleen manifested pronounced immunosuppression and compromised protein processing during the immune response, despite gradual recovery of mitochondrial ATP synthesis and ribosomal assembly. These findings implicate Kupffer cells in triggering hepatic injury repair mechanisms during bacterial clearance (Zhao et al., 2025), whereas continuous splenic lymphocyte depletion may have severely impaired adaptive immune responses (Saeidi et al., 2018). Notably, splenic damage inflicted by APEC infection requires a longer recovery period than hepatic injury.

Network analysis of hepatic DEGs identified the MCM complex, *CDT1*, and *CDK2* as pivotal regulators of cell proliferation, mediating structural repair. Previous studies have reported that pathogen infection disrupts host cell-cycle progression by targeting DNA replication licensing and *CDK*-dependent checkpoints. Dysregulation of the MCM complex, *CDT1*, and *CDK2* leads to altered S-phase entry and proliferation capacity, thereby reshaping immune cell activation and tissue repair responses during infection (Sekelova et al., 2017; Wang et al., 2016). *IL1B* within the C-type lectin receptor signaling pathway emerged as a critical modulator of immune responses in both the liver and the spleen, while *PTGS2* and *NFKBIA* constitute key inflammatory regulators in the spleen. *IL1B* is a key proinflammatory cytokine that is rapidly upregulated upon pathogen infection and amplifies innate immune responses (Corcoran and O'Neill, 2016). Chen et al. (2025b) reported that modulation of NF-κB signaling, characterized by suppression of *PTGS2* and concomitant upregulation of *NFKBIA*, effectively attenuated TNF-α-induced inflammatory responses, underscoring the critical immunoregulatory role of this gene expression pattern (Reuben et al., 2021). These observations align with the cooperative “liver–spleen axis” paradigm in host defense and underscore a conserved, dynamically regulated gene expression mechanism underlying systemic infection responses (Fonseca et al., 2021).

This study profiled the systemic responses of chicken liver and spleen to APEC infection, offering insights into host immunity and resistance. However, several limitations warrant consideration. The temporal boundaries between the acute infection and recovery phases remain poorly defined, with substantial overlap among them. While increased sampling frequency would better resolve the kinetics of host immune responses, unpredictable post-infection mortality and inter-individual variation in pathogen defense necessitate larger cohorts. Furthermore, the inability to prospectively determine the survival outcomes of broilers sampled at specific time points further underscores this necessity. Therefore, future studies should use larger sample sizes and higher sampling frequency to refine this dynamic model of APEC pathogenesis and host recovery.

## Conclusion

Broilers mounted divergent immune responses in the liver and spleen during APEC infection. The liver rapidly cleared pathogens, restored protein synthesis, and initiated cell proliferation, whereas the spleen exhibited sustained cellular damage, persistent inflammation, and ferroptosis activation. Our findings demonstrate that host resistance is orchestrated through multi-gene interactions and inter-organ coordination.

## CRediT authorship contribution statement

**Tailong Wang:** Writing – review & editing, Writing – original draft, Methodology, Investigation, Formal analysis, Data curation. **Yifei He:** Methodology, Investigation. **Tingyu Zhang:** Methodology, Investigation. **Yan Luo:** Resources, Methodology, Investigation. **Mengru Chen:** Methodology, Investigation. **Chu Meng:** Methodology, Investigation. **Haitao Zhang:** Resources, Methodology, Investigation. **Ruqian Zhao:** Writing – review & editing, Resources, Project administration, Conceptualization. **Yimin Jia:** Writing – review & editing, Resources, Project administration.

## Disclosures

This manuscript is an original, unpublished work and has not been submitted to any other journals for publication, in whole or in part. All authors listed have read and approved this manuscript, and there is no conflict of interest in the submission of this manuscript. The authors declare that they have no known competing financial interests or personal relationships that could have appeared to influence the work reported in this paper.

## Appendix Supplementary materials

Image, application 1

Image, image 2 dummy alt text

Image, image 3 dummy alt text

Image, application 4

Image, application 5
