# Supplementary material for: Transcription profiles of chicken liver and spleen in response to infection with avian pathogenic Escherichia coli at different stages
Source: Poult Sci. 2026 Feb 2;105(5):106579. doi: 10.1016/j.psj.2026.106579 (PMC12917521; doi:10.1016/j.psj.2026.106579)
Supplement: Supplementary file 1 [file mmc1.pdf]

EditorBar 北京艾迪润色

EditorBar Language Editing
No. 35, Tsinghua East Road, Beijing, China 100083
Email: runse@editorbar.com Phone: +86-10-5620-8614

CERTIFICATE OF LANGUAGE EDITING

The English writing of the following manuscript was carefully edited by a native English speaker.

Manuscript Information

ID	LE202601210148
Editing date	2026-01-24
Title	Transcription profiles of chicken liver and spleen in response to infection with avian pathogenic Escherichia coli at different stages
Corresponding author	Yimin Jia
Language writing before editing	□ Very poor □ Poor ■ Fair □ Good □ Very good □ Excellent
Recommendation after language editing	□ Submitting to target journal directly<br>■ Submitting to target journal after minor revision<br>□ Re-editing required after major revision<br>□ Not suitable for publication
Overview comments	Dear Authors, Thank you for the opportunity to assist you with your interesting manuscript, titled “Transcription profiles of chicken liver and spleen at various stages following infection with avian pathogenic Escherichia coli.” As requested, I have edited the text for language, grammar, and punctuation, and have improved its clarity. I used American spelling when editing, as this was consistent with what you used in the manuscript. If British spelling is desired, some words will be spelled differently. I have inserted several comments with suggestions for your consideration throughout the text. The abstract did not mention the age of the broilers used in this study. I recommend adding this information. Please note that abbreviations should be defined upon their first use in the abstract, the main text, and the legend for each figure/table. Once defined, the abbreviation should be used consistently and should not be redefined in the same article component. The guiding rule is to keep the number of abbreviations to a minimum—ideally under ten in the main text—and use them only if they help the readers (I would avoid abbreviating single words like glucose, albumin, etc.). Those not used in the text or used fewer than 3–4 times should not be defined (unless they abbreviate a very long/complicated term). I recommend reviewing the manuscript to remove rarely or never used abbreviations and to ensure that those requiring definition (including those used in the tables and figures) are properly defined. Standard nomenclature dictates that official gene symbols (including mRNA) should be italicized, while their corresponding protein symbols should not. This rule only applies to official symbols. In birds, both official protein and gene symbols are written in uppercase letters. I italicized gene symbols whenever italization was missing and removed italization when the abbreviated name was not an official symbol (e.g., contained a hyphen or a Greek letter). I strongly recommend that you review all gene and protein names to ensure accurate formatting. You used some tools, software programs, etc. For some, you have indicated the source by naming of the maker. Yet, for some, there is no indication of the source. I recommend reviewing the article and ensuring that all makers are credited with their full names and location details (city, state/province, if relevant, and country). Whenever reporting a p-value in the Results section, I recommend also reporting the values being compared. I also suggest reporting exact p values whenever possible. The Results section reports findings from tests not described in the Methods section (e.g., assessments beyond 5 dpi and apoptosis). This needs to be corrected; all experiments, tests, and assessments must be thoroughly described in the Methods section. The Results section contains information already described in the Methods section, and interpretations of findings that belong in the Discussion section. The Results section should objectively present

your findings—and nothing more. I used comments to point out several examples (although I did not mark all of them). Please review this section and move or remove any text that does not report a result of your study. The section also reports results of experiments that were not described in the Methods section. This should be corrected. I recommend using the first paragraph of the Discussion section to present a brief summary of your most important or interesting results, and associate these with the study's objectives and hypothesis outlined in the Introduction section. The Discussion and Conclusion sections should not present any new results (e.g., ferroptosis). Anything related to your study should be described in the Methods and Results sections before it is discussed in the Discussion and Conclusion sections. I recommend adding a sentence to your Conclusion section that connects your findings and conclusions with the study hypothesis stated in the Introduction, indicating whether your findings support your hypothesis. Should you have any concerns, please feel free to get back to me. I wish you the best of success with your manuscript.

Edited by

Joseph S.

Senior Editor
The Hebrew University of Jerusalem/The University of Teramo
Zoology

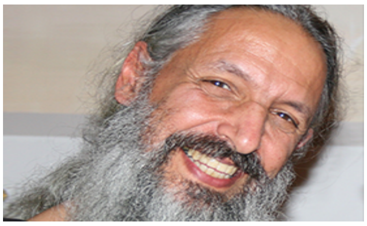

Certificate Issued by

Dr. Jason Qee

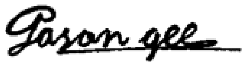

Editor in Chief
Editorbar Language Editing, Beijing, China
runse@editorbar.com www.editorbar.com

Certificate link: www.editorbar.com/order/cert/LE202601210148
