# Supplementary figures and images for: Transcription profiles of chicken liver and spleen in response to infection with avian pathogenic Escherichia coli at different stages
Source: Poult Sci. 2026 Feb 2;105(5):106579. doi: 10.1016/j.psj.2026.106579 (PMC12917521; doi:10.1016/j.psj.2026.106579)

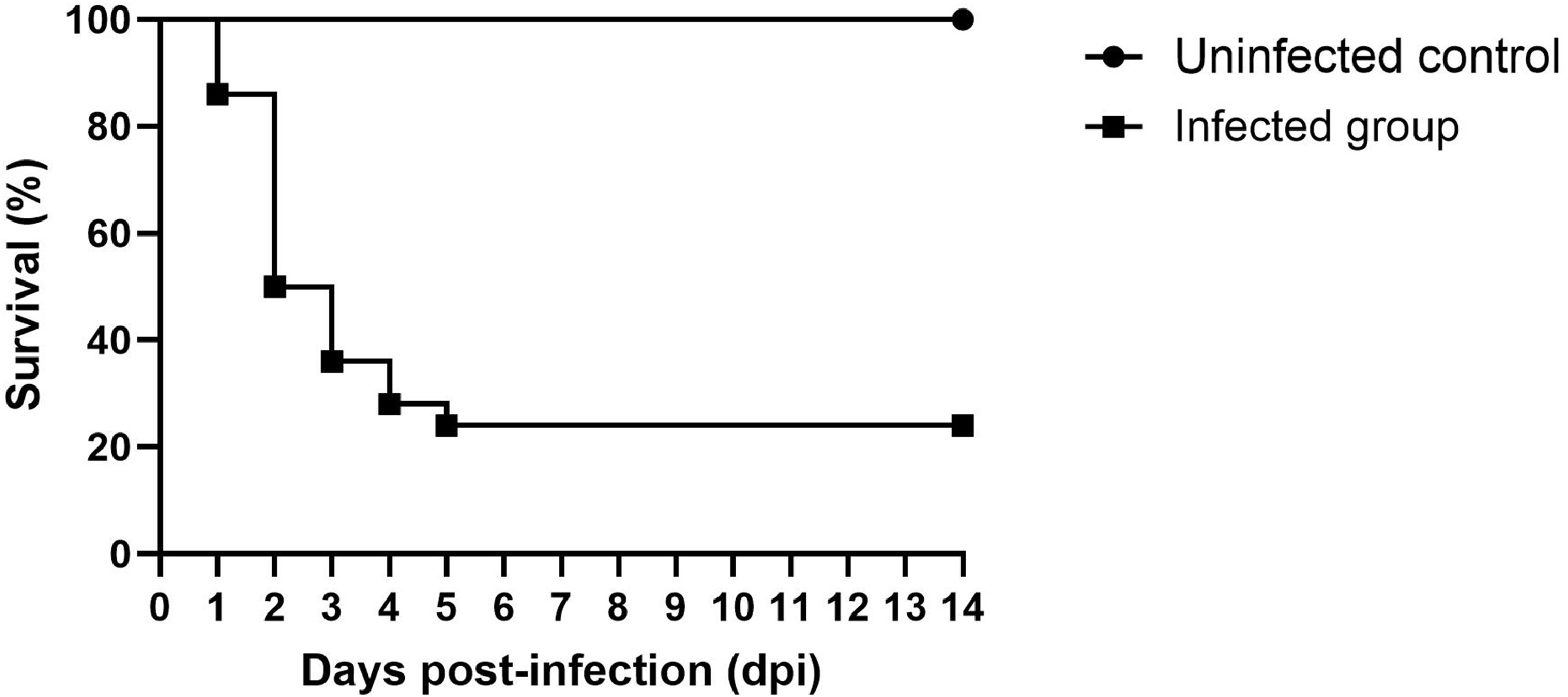

Supplement: Supplementary file 2 [file mmc2.jpg]

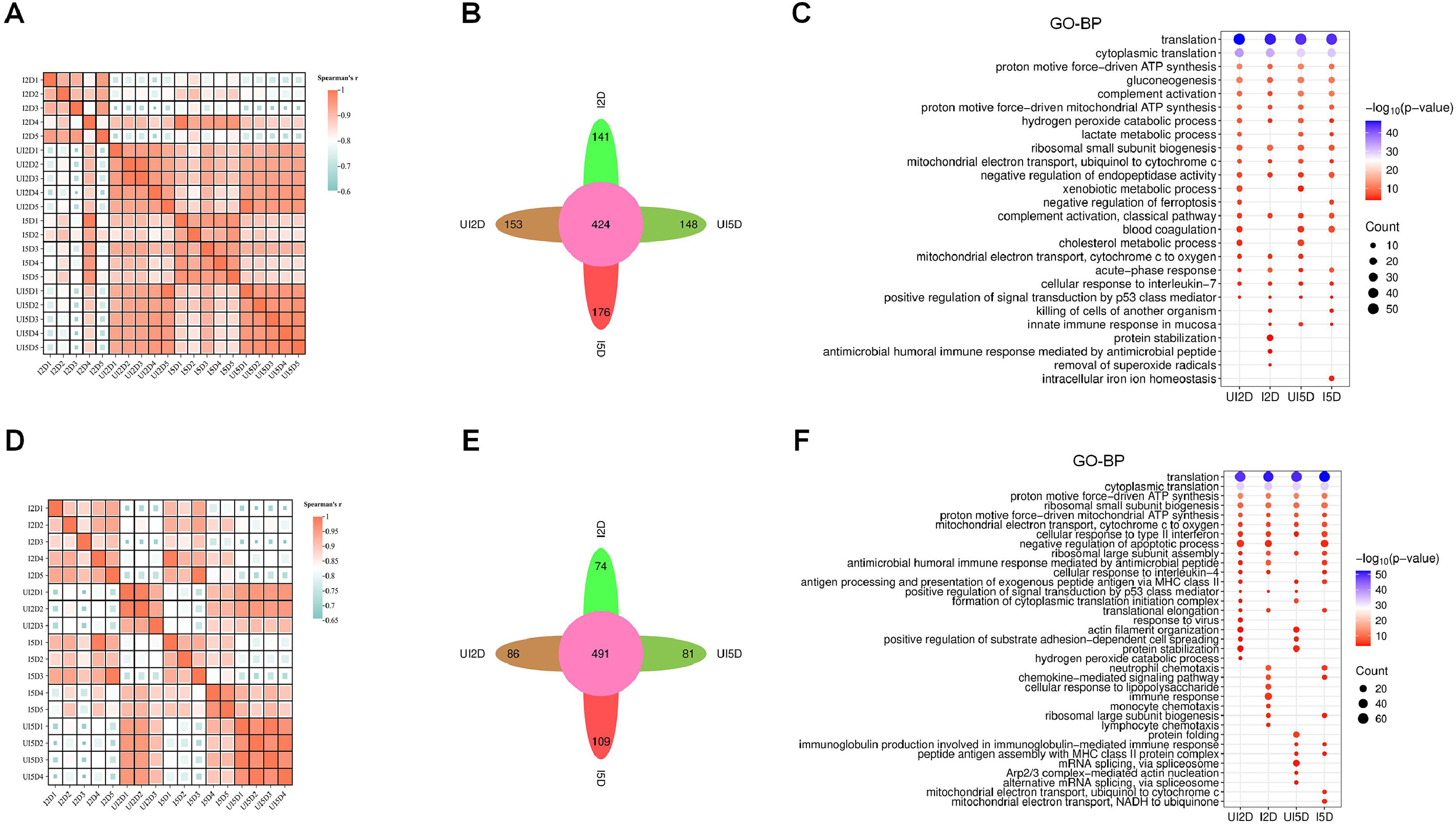

Supplement: Supplementary file 3 [file mmc3.jpg]
