# Supplementary material for: Transcription profiles of chicken liver and spleen in response to infection with avian pathogenic Escherichia coli at different stages
Source: Poult Sci. 2026 Feb 2;105(5):106579. doi: 10.1016/j.psj.2026.106579 (PMC12917521; doi:10.1016/j.psj.2026.106579)
Supplement: Supplementary file 4 [file mmc4.docx]

**Supplementary Figure 1.** Kaplan-Meier survival curve for uninfected control and infected groups of broilers challenged with APEC.

The Kaplan-Meier survival curves depict the survival probabilities over a 14-day period for uninfected control (circle-marked line) and infected (square-marked line) groups of broilers (n=50 per group). The y-axis shows survival probability, and the x-axis indicates dpi.

**Supplementary Figure 2.** Expression profiles of liver and spleen in broilers challenged with APEC.

(A) Spearman’s r heatmap for gene expression profiles of the 20 liver samples; (B) Flower plot of the top 5% most highly expressed genes at liver. The peripheral numbes represent the counts of group-specific genes, and the central number represents the number of co-expressed genes across all group; (C) The top 20 significantly enriched Gene Ontology-Biological Process (GO-BP) terms of the top 5% most highly expressed genes at liver; (D) Spearman’s r heatmap for gene expression profiles of the 17 spleen samples; (E) Flower plot of the top 5% most highly expressed genes at spleen; (F) The top 20 significantly enriched Gene Ontology-Biological Process (GO-BP) terms of the top 5% most highly expressed genes at spleen.
