# Supplementary material for: Transcription profiles of chicken liver and spleen in response to infection with avian pathogenic Escherichia coli at different stages
Source: Poult Sci. 2026 Feb 2;105(5):106579. doi: 10.1016/j.psj.2026.106579 (PMC12917521; doi:10.1016/j.psj.2026.106579)
Supplement: Supplementary file 5 [file mmc5.docx]

**Table S1** Expression and functional pathway analysis of liver candidate genes.

| Genes | 2d log2FC | 5d log2FC | KEGG pathway |
| --- | --- | --- | --- |
| IL1β | 5.56 | 1.11 | C-type lectin receptor signaling pathway |
| CDT1 | -1.65 | NS | Cell cycle |
| CDK1 | NS | 1.34 | Cell cycle |
| CDK2 | -1.56 | NS | Cell cycle |
| CDC45 | -1.66 | NS | Cell cycle |
| MCM2 | -2.17 | NS | Cell cycle/DNA replication |
| MCM3 | -2.62 | 1.18 | Cell cycle/DNA replication |
| MCM4 | -2.00 | NS | Cell cycle/DNA replication |
| MCM5 | -2.37 | 1.09 | Cell cycle/DNA replication |
| MCM6 | -1.94 | NS | Cell cycle/DNA replication |

Note: 2d log2FC, [log2 (fold change) at 2 days post-infection]; 5d log2FC, [log2 (fold change) at 5 days post-infection].

**Table S2** Expression and functional pathway analysis of splenic candidate genes.

| Genes | 2d log2FC | 5d log2FC | KEGG pathway |
| --- | --- | --- | --- |
| GPX4 | NS | 1.18 | Ferroptosis |
| GPX7 | -1.77 | NS | Glutathione metabolism |
| IL1β | 4.20 | 3.56 | MAPK signaling pathway/Herpes simplex virus 1 infection/C-type lectin receptor signaling pathway |
| IL6 | 4.55 | 4.44 | Herpes simplex virus 1 infection/C-type lectin receptor signaling pathway |
| JUN | 1.16 | 1.03 | MAPK signaling pathway/C-type lectin receptor signaling pathway/Apoptosis |
| FOS | 3.96 | 2.62 | C-type lectin receptor signaling pathway/MAPK signaling pathway/Apoptosis |
| PTGS2 | 1.47 | NS | Efferocytosis/C-type lectin receptor signaling pathway |
| NFKBIA | 1.01 | NS | Herpes simplex virus 1 infection/C-type lectin receptor signaling pathway/Apoptosis/Adipocytokine signaling pathway/RIG-I-like receptor signaling pathway |
| CYSLTR2 | -2.20 | -1.11 | Neuroactive ligand-receptor interaction |
| HPGDS | 2.72 | 4.10 | Drug metabolism - cytochrome P450/Metabolism of xenobiotics by cytochrome P450 |

Note: 2d log2FC, [log2 (fold change) at 2 days post-infection]; 5d log2FC, [log2 (fold change) at 5 days post-infection].
